# Punicalagin Protects Ram Sperm from Oxidative Stress by Enhancing Antioxidant Capacity and Mitochondrial Potential during Liquid Storage at 4 °C

**DOI:** 10.3390/ani14020318

**Published:** 2024-01-19

**Authors:** Liuming Zhang, Xuyang Wang, Tariq Sohail, Caiyu Jiang, Yuxuan Sun, Jian Wang, Xiaomei Sun, Yongjun Li

**Affiliations:** Key Laboratory for Animal Genetics and Molecular Breeding of Jiangsu Province, College of Animal Science and Technology, Yangzhou University, Yangzhou 225009, China; 18352767281@163.com (L.Z.); 18705271593@163.com (X.W.); drtariqsohail34@yahoo.com (T.S.); m19352674787@163.com (C.J.); 19802622001@163.com (Y.S.); jianwang1223@163.com (J.W.); xiaomeisun_yz@163.com (X.S.)

**Keywords:** punicalagin, ram, sperm, antioxidant capacity, 4 °C preservation

## Abstract

**Simple Summary:**

Ram sperm is highly sensitive to reactive oxygen species (ROS) during liquid storage at 4 °C. Diluent supplementation with an antioxidant could help protect sperm from oxidative stress. Punicalagin, the primary compound in pomegranate extract, is a polyphenol with potent antioxidant properties and has the ability to scavenge free radicals like DPPH. However, the impact of punicalagin on *Hu* ram sperm during liquid storage at 4 °C is unknown. Therefore, different concentrations (0, 5, 15, 30 and 45 μM) of punicalagin were added to *Hu* ram semen preserved at 4 °C to explore the effect on sperm. The results indicated that the addition of punicalagin to the diluent could enhance the quality of ram sperm preserved at 4 °C by increasing antioxidant capacity, mitochondrial potential and reducing oxidative stress. Furthermore, 30 μM of punicalagin was the optimal concentration for *Hu* ram sperm preserved at 4 °C.

**Abstract:**

The aim of this study was to investigate the effect of punicalagin, an antioxidant, on ram sperm quality. Semen samples were collected and pooled from five rams, then diluted using a Tris-based diluent containing various concentrations (0, 5, 15, 30 and 45 μM) of punicalagin. Sperm motility, plasma membrane integrity, acrosome integrity, total antioxidant capacity (TAC), reactive oxygen species (ROS), malondialdehyde (MDA), mitochondrial membrane potential (MMP), superoxide dismutase (SOD) and catalase (CAT) were measured and analyzed during liquid storage at 4 °C. The results showed that the Tris-based solution containing punicalagin improved sperm motility, plasma membrane integrity, acrosome integrity, TAC, SOD, CAT and MMP, and decreased ROS content and MDA content. At the same time, the semen sample diluted with the Tris-based solution supplemented with 30 μM punicalagin achieved the best effect. The sperm total motility, progressive motility, plasma membrane integrity, acrosome integrity, TAC, SOD, CAT and MMP of the group supplemented with 30 μM punicalagin were significantly (*p* < 0.05) higher than those of the other groups on the 5th day during the liquid storage at 4 °C. Meanwhile, the ROS content and MDA content were significantly (*p* < 0.05) lower than those in the other groups. In conclusion, the optimal concentration of punicalagin in the *Hu* ram semen diluent was determined to be 30 μM. The results indicated that a diluent supplemented with punicalagin could enhance the quality of ram sperm preserved at 4 °C by increasing antioxidant capacity, mitochondrial potential and reducing oxidative stress.

## 1. Introduction

In the sheep industry, artificial insemination (AI) is commonly performed using ram semen during liquid storage at room temperature and 4 °C [1]. Therefore, effective long-term storage of functional ram sperm is also an important target for the sheep industry [2]. However, ram sperm exhibit morphological and functional changes, such as decreased motility and acrosome integrity after liquid storage. Maintaining high sperm motility and acrosome integrity is crucial for sperm to penetrate the zona pellucida and corona radiata, enabling them to successfully bind with oocytes and achieve fertilization [3,4,5]. This result could be improved by adding external substances to enhance semen preservation [6].

Energy metabolism is essential for all cells to maintain normal physiological function [7]. Spermatozoa are highly specialized cells that differ significantly from non-spermatozoa in morphological structure. During spermatogenesis, most of the organelles are discarded. Only a small number of closely related organelles with similar functions remain. The mitochondria are important organelles for spermatozoa. The energy metabolic pathway for spermatozoa to produce adenosine triphosphate (ATP) consists of glycolysis and mitochondrial oxidative phosphorylation (OXPHOS) [8]. Enzymes involved in glycolysis are primarily located in the fibrous sheath of the sperm tail [9]. OXPHOS mainly occurs in the mitochondria of the mid-piece of the sperm [10]. Compared to glycolysis, mitochondrial OXPHOS is a more efficient energy metabolic pathway for producing ATP in sperm [11,12]. Therefore, normal functioning mitochondria play a crucial role in sperm quality.

However, the process of OXPHOS in mitochondria can produce reactive oxygen species (ROS) [13,14]. Physiological levels of ROS will not damage sperm, but excessive ROS can lead to oxidative stress and damage sperm function [15,16]. As semen preservation time is extended, mitochondria will produce more ROS. The antioxidant capacity of sperm is insufficient to counteract the excessive ROS, leading to lipid peroxidation (LPO) in the mid-section of the sperm tail and resulting in mitochondrial damage [17,18]. After excessive ROS cause mitochondrial damage, the mitochondria will increase the production of ROS, leading to further damage to the mitochondria, thus perpetuating the cycle [19]. This affects the function of mitochondria, damages the structure of sperm, and ultimately reduces the efficacy of semen preservation.

Punicalagin is a natural phenolic compound extracted from pomegranate, known for having the largest molecular weight among phenolic compounds [20,21]. Punicalagin has numerous biological functions, such as protecting the kidneys, inhibiting the growth of liver cancer cells and exerting a beneficial protective effect on the reproductive system [22,23,24]. In a study involving rats, Sudheesh [25] found that the content of malondialdehyde (MDA) in the liver decreased significantly, while the activities of enzymes such as catalase (CAT), superoxide dismutase (SOD) and glutathione (GSH) significantly increased when the rats were fed at a dose of 10 mg/day. Cayir [26] discovered that punicalagin can also neutralize free radicals such as O^2−^, enhance the activities of antioxidant enzymes such as SOD and ultimately mitigate the damage to the liver and kidneys caused by oxidative stress. Punicalagin, the primary compound in pomegranate extract, is a polyphenol with potent antioxidant properties and the ability to scavenge free radicals like DPPH [27].

However, the effect of punicalagin on ram sperm quality is unknown. In the present study, we aimed to verify whether punicalagin could protect ram sperm during liquid storage at 4 °C and whether punicalagin’s regulation of oxidative stress is applicable to ram sperm. Therefore, the purpose of this study is to explore whether punicalagin can enhance the antioxidant capacity of ram sperm, maintain the normal function of sperm mitochondria and ultimately improve the quality of semen preservation by assessing oxidative stress parameters, mitochondrial membrane potential (MMP), functional integrity and motility parameters.

## 2. Materials and Method

### 2.1. Animals and Semen Collection

All experimental procedures involving rams were conducted in accordance with the standard guidelines of the Animal Care Committee of the Yangzhou University (Approval ID: 202206132). Compared to other breeds, *Hu* sheep are popular among farms due to their high fertility, multiple births and estrus all year round. Healthy breeding *Hu* rams (*n* = 5), with an average of 1.5–2.5 years, body weight 75 ± 5 kg with good libido had their semen collected using an artificial vagina. The rams were fed 500 g concentrate/day, as well as hay and free water at the Yangzhou University Experimental Ranch. Semen samples were collected twice per week for four consecutive months (from March to July 2023). A total of 160 ejaculates (32 ejaculates per ram) were used for the experiments. Collected semen samples were taken back to the laboratory within 20 min of staining at 37 °C and the sperm quality was measured using a computer-assisted sperm analyzer (CASA, ML-608JZ II, Mailang, Nanning, China). Ejaculates with volume > 0.6 mL, total motility > 80% (CASA detection), total abnormality < 10% (Gentian violet staining) and the concentration > 2.0 × 10^9^ sperm/mL (CASA detection) were pooled to eliminate individual differences for the further experiment.

### 2.2. Diluent Preparation

In the present experiment, the Tris-based (Tris 3.07 g, fructose 2 g, citric acid 1.64 g, 50,000 IU penicillin, 50,000 IU streptomycin, 0.1 g soy lecithin and distilled water up to 100 mL) semen basic diluent was used. Punicalagin (HPLC ≥ 98%, Mansite, Chengdu, China) was dissolved in water and added to the basic diluent at concentrations of 5, 15, 30 and 45 µM, while the control was the basic extender without ALA. The osmolality and pH of the diluent were 350 mOsm/L and 7.4.

### 2.3. Semen Processing

After evaluating the quality of the semen samples, the qualified semen samples were pooled and divided into five tubes. One tube was diluted with a basic diluent to serve as the control group. The other tubes were diluted with a diluent of varying concentrations (containing 5, 15, 30, 45 μM punicalagin) and all the tubes were diluted to 2 × 10^8^ sperm/mL. Finally, semen samples were wrapped in a towel and stored in the refrigerator at 4 °C. During the preservation period, semen quality parameters such as total motility (TM, %), progressive motility (PM, %), straight line velocity (VSL, µm/s), curvilinear velocity (VCL, µm/s), average path velocity (VAP, µm/s), average motion degree (MAD, ◦/s), plasma membrane and acrosome integrity were measured and analyzed once a day. Sperm antioxidant capacity such as ROS, MDA, total antioxidant capacity (TAC), SOD and CAT was measured and analyzed on the first, third and fifth day of semen preservation. The sperm MMP was evaluated on the first, third and fifth day during the preservation period. All experiments were performed with six replicates for both the control and treatment groups.

### 2.4. Semen Quality Evaluation

#### 2.4.1. Analysis of Sperm Motility Parameters

The sperm motility parameters (TM, PM, VSL, VCL, VAP and MAD) were measured using the CASA. The diluents and counting chambers used in CASA detection have a significant impact on the accuracy and stability of the results [28]. Preserved semen samples were diluted to 4 × 10^7^ sperm/mL using a basic diluent and then incubated at 37 °C for 3 min. A total of 1.4 µL semen sample was dropped on a MACRO sperm counting chamber (YA-1, 10 µm, Yucheng, Nanjing, China) and then positioned on a 37 °C stage warmer using a phase-contrast microscope (ML-800, Mailang, Nanning, China) at a magnification of 100×, equipped with a CCD-camera (MD06200C, Mailang, Nanning, China) for the measurement. A total of at least 1000 sperm in five fields for each sample were counted using the CASA.

#### 2.4.2. Analysis of Sperm Plasma Membrane Integrity

The percentage of hypotonic swelling test (HOST)-positive sperm was evaluated to analyze the functional integrity of the sperm plasma membrane, as described by Vasquez [29]. The preserved semen sample was individually incubated with a fresh hypotonic swelling solution (consisting of 0.49 g sodium citrate and 0.9 g fructose dissolved in 100 mL distilled water, 108 mOsm/L) at a ratio of 1:10 at 37 °C for 30 min. After incubation, 1.4 µL of the sample was dropped on a specialized sperm counting plate. The percentage of coiling sperm was counted using a phase-contrast microscope (OLYMPUS CX31, Tokyo, Japan) at a magnification of 400×, with at least 200 sperm being counted.

#### 2.4.3. Analysis of Sperm Acrosome Integrity

The functional integrity of the sperm acrosome was assessed using Coomassie brilliant blue staining as described by Wang [30]. A Coomassie brilliant blue solution was prepared by first dissolving 0.1 g G-250 in 50 mL of 95% absolute ethanol. Secondly, 100 mL of 85% phosphoric acid was added. Finally, distilled water was added to make the total volume up to 1000 mL. After staining with Coomassie brilliant blue for 30 min at room temperature, each sample was examined under a phase-contrast microscope (OLYMPUS CX31, Tokyo, Japan) at a magnification of 1000× and at least 200 sperm were counted. The head of the intact acrosome sperm was stained blue.

#### 2.4.4. Analysis of Sperm Antioxidant Capacity

ROS content, MDA content and SOD activity were measured to assess the antioxidant capacity of the sample using kits (The Beyotime Institute of Biotechnology, Shanghai, China) according to the manufacturer’s instructions. ROS content was measured using DCFH-DA. Briefly, preserved semen samples were centrifuged to remove the supernatant. The samples were resuspended in PBS to a concentration 4 × 10^7^ sperm/mL. A total of 300 µL DCFH-DA working solution was added to the 50 µL semen sample and then incubated at 37 °C for 30 min in the dark. The multifunctional microplate reader (PerkinElmer, Waltham, MA, USA) was used to measure the fluorescence intensity at excitation/emission = 488/525 nm. The fluorescence intensity is commonly used to represent the ROS content. MDA content was measured using TBA. Briefly, preserved semen samples were centrifuged to get the supernatant. A total of 200 µL TBA working solution was added to the 100 µL supernatant and then heated at 100 °C for 15 min. After staining, the samples were centrifuged at 1000× *g* for 10 min. The multifunctional microplate reader (PerkinElmer, Waltham, MA, USA) was used to measure the absorbance at 532 nm. The MDA content can be determined by substituting the absorbance into the standard curve. SOD activity was measured using WST-8. Briefly, preserved semen samples were centrifuged to remove the supernatant. The samples were lysed using the lysis buffer provided in the kit. The protein concentration was measured using the BCA Protein Assay Kit (Solarbio Institute, Beijing China) according to the manufacturer’s instructions. The working solution was added to the lysed sample and incubated at 37 °C for 30 min. The multifunctional microplate reader (PerkinElmer, Waltham, USA) was used to measure the absorbance at 450 nm. The SOD activity can be determined by substituting the absorbance into the standard curve.

CAT activity and TAC were measured to determine the antioxidant capacity in sperm using kits (Nanjing Jiancheng Bioengineering Institute, Nanjing, China) according to the manufacturer’s instructions. Briefly, preserved semen samples were centrifuged to get the supernatant. The working solution was added to the supernatant according to the manufacturer’s instructions. The multifunctional microplate reader (PerkinElmer, Waltham, USA) was used to measure the absorbance at 405 nm. The CAT activity and TAC can be obtained by substituting the absorbance into the standard curve.

#### 2.4.5. Analysis of Sperm MMP

MMP was measured using an MMP assay kit with JC-1 (The Beyotime Institute of Biotechnology, Shanghai, China) according to the manufacturer’s instructions. The orange fluorescence was used to represent high MMP when JC-1 formed multimeric aggregates were excited, while green indicated the opposite. Briefly, preserved semen samples were centrifuged to remove the supernatant. The samples were resuspended in PBS to a concentration 4 × 10^7^ sperm/mL. A total of 3 µL JC-1 (200×) was added to the 100 µL semen sample and then incubated at 37 °C for 15 min in the dark. The multifunctional microplate reader (PerkinElmer, Waltham, USA) was used to measure the orange fluorescence at excitation/emission = 525/590 nm and the green fluorescence at excitation/emission = 488/525 nm. The relative ratio of orange–green fluorescence is commonly used to represent the MMP.

### 2.5. Statistical Analysis

All the data were analyzed using the Statistical Package for the Social Sciences (SPSS, IBM, Armonk, NY, USA, version 25.0). The Shapiro–Wilk test was used to assess the normality of the data. The data showed a normal distribution. The parameters were evaluated and analyzed using a two-way repeated measures ANOVA. Data were presented as the “mean ± SEM” and a *p* value of <0.05 (*p* < 0.05) was considered significant. All experiments were performed with six replicates for both the control and treatment groups.

## 3. Results

### 3.1. Sperm Motility Parameters

The effects of different concentrations of punicalagin on *Hu* ram sperm motility at 4 °C are shown in Table 1. Sperm TM and PM were decreased with the advancement in time. The addition of punicalagin improved the sperm TM and PM during liquid storage from the 1st day to the 5th day. The TM and PM of the 5 μM treatment group were not significantly (*p* > 0.05) higher than those of the control group on the 2nd day. In the other treatment groups the sperm TM and PM were significantly (*p* < 0.05) improved from the 2nd day to the 5th day. The sperm TM and PM of the 30 μM treatment group were the highest (*p* < 0.05) compared to those of the control group from the 2nd day to the 5th day.

### 3.2. Sperm Kinematic Parameters

As shown in Table 2, the sperm VSL, VCL, VAP and MAD gradually decreased over time. On the 2nd and 4th days, the sperm VSL of the 30 μM treatment group was significantly (*p* < 0.05) higher than that of the other groups. On the 3rd day, the sperm VCL of the 30 μM treatment group was significantly (*p* < 0.05) higher than that of the other groups. On the 4th and 5th days, the sperm VCL of the 30 μM treatment group was significantly (*p* < 0.05) higher than that of the control group. The sperm VAP of the 30 μM treatment group was significantly (*p* < 0.05) higher than that of the control group from the 3rd day to the 5th day. The sperm MAD of the 30 μM treatment group was significantly (*p* < 0.05) higher than that of the control group from the 1st day to the 3rd day. On the 5th day, all the treatment groups were significantly (*p* < 0.05) higher than that of the control group.

### 3.3. Sperm Plasma Membrane and Acrosome Integrity

The effects of different concentrations of punicalagin on *Hu* ram sperm plasma membrane and acrosome integrity during liquid storage at 4 °C are shown in Table 3. On the 1st day, the sperm plasma membrane of the 30 μM treatment group was significantly (*p* < 0.05) higher than that of the control, 5 and 45 μM groups and was also higher (*p* > 0.05) than that of the 15 μM group. The sperm plasma membrane of the 30 μM treatment group was significantly (*p* < 0.05) higher than that of the other groups from the 2nd day to the 5th day. On the 1st day, the sperm acrosome integrity of the 30 μM treatment group was significantly (*p* < 0.05) higher than that of the control and 5 μM groups, and it was also higher (*p* > 0.05) than that of the 15 and 45 μM groups.

### 3.4. Sperm TAC Activity, ROS Content and MDA Content

**Figure 1:**
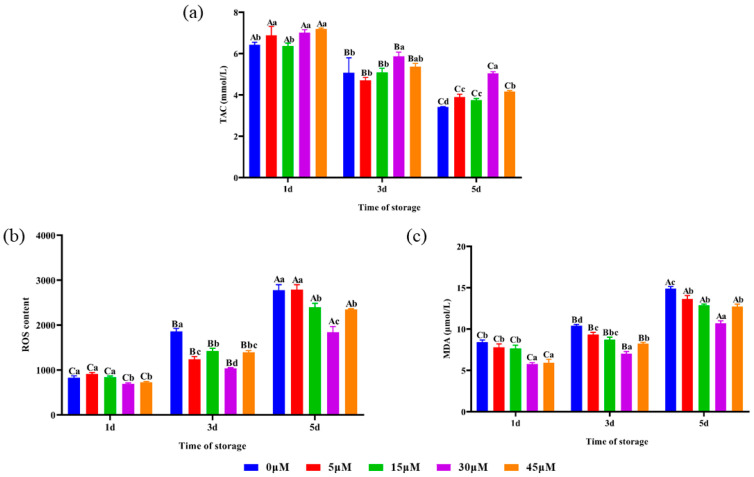
Effects of different concentrations of punicalagin on TAC activity, ROS content and MDA content of *Hu* ram semen preserved at 4 °C. (**a**) Sperm TAC activity, (**b**) sperm ROS content and (**c**) sperm MDA content. Different lowercase letters above their bars indicate significant (*p* < 0.05) differences among different concentration treatment groups on the same day. Different capital letters above their bars indicate significant (*p* < 0.05) differences among different days in the same concentration group.

The effects of different concentrations of punicalagin on *Hu* ram sperm TAC activity during liquid storage at 4 °C are shown in Figure 1a. On the 3rd day, the sperm TAC activity of the 30 μM treatment group was significantly (*p* < 0.05) higher than that of the control, 5 and 15 μM groups and was also higher (*p* > 0.05) than that of the 45 μM group. On the 5th day, the sperm TAC activity of the 30 μM treatment group was significantly (*p* < 0.05) higher than that of the other groups. As shown in Figure 1b, the sperm ROS content of the 30 and 45 μM treatment groups was significantly (*p* < 0.05) lower than that of the control, 5 and 15 μM groups on the 1st day. On the 3rd and 5th days, the sperm ROS content of the 30 μM treatment group was significantly (*p* < 0.05) lower than that of the other groups. As shown in Figure 1c, the sperm MDA content of the 30 and 45 μM treatment groups was significantly (*p* < 0.05) lower than that of the control, 5 and 15 μM groups on the 1st day. On the 3rd and 5th days, the sperm MDA content of the 30 μM treatment group was significantly (*p* < 0.05) lower than that of the other groups.

### 3.5. Sperm MMP, SOD Activity and CAT Activity

**Figure 2:**
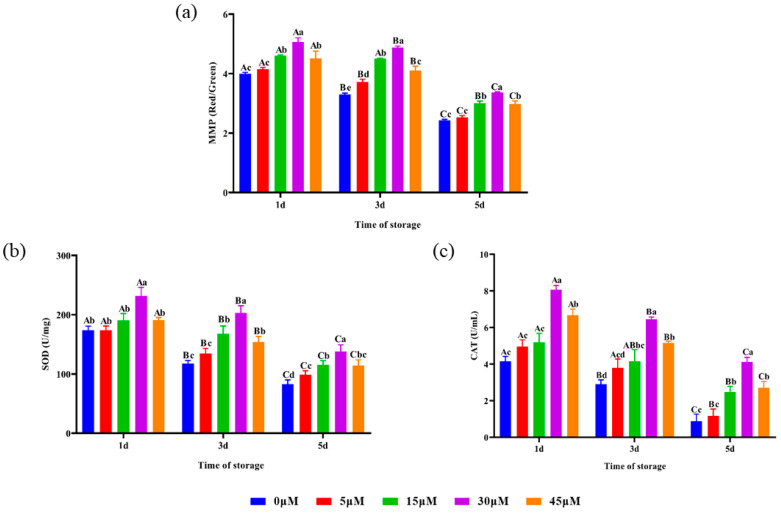
Effects of different concentrations of punicalagin on MMP, SOD activity and CAT activity of *Hu* ram semen preserved at 4 °C. (**a**) Sperm TAC activity, (**b**) sperm ROS content and (**c**) sperm MDA content. Different lowercase letters above their bars indicate significant (*p* < 0.05) differences among different concentration treatment groups on the same day. Different capital letters above their bars indicate significant (*p* < 0.05) differences among different days in the same concentration group.

The effects of different concentrations of punicalagin on *Hu* ram sperm MMP during liquid storage at 4 °C are shown in Figure 2a. The sperm MMP of the 15, 30 and 45 μM treatment groups was significantly (*p* < 0.05) higher than that of the control and 5 μM groups and the sperm MMP of the 30 μM treatment group was significantly (*p* < 0.05) higher than that of the other groups on the 1st, 3rd and 5th days. As shown in Figure 2b, the sperm SOD activity of the 30 μM treatment group was significantly (*p* < 0.05) higher than that of the other groups on the 1st day. On the 3rd and 5th days, the sperm SOD activity of the 30 μM treatment group was significantly (*p* < 0.05) higher than that of the other groups. As shown in Figure 2c, the sperm CAT activity of the 30 and 45 μM treatment groups was significantly (*p* < 0.05) higher than that of the control, 5 and 15 μM groups on the 1st day. On the 3rd and 5th days, the sperm CAT activity of the 15, 30 and 45 μM groups was significantly (*p* < 0.05) higher than that of the control group. On the 5th day, the sperm CAT activity of the 15, 30 and 45 μM groups was significantly (*p* < 0.05) higher than that of the control group. On the 1st, 3rd and 5th days, the sperm CAT activity of the 30 μM treatment group was significantly (*p* < 0.05) higher than that of the other groups.

## 4. Discussion

Semen diluents without antioxidants cannot maintain the quality of ram semen due to the excessive production of ROS during liquid storage [31]. Adding an appropriate antioxidant to the diluent could help maintain sperm function and counteract the effects of oxidative stress. Punicalagin is a free radical scavenger with potent antioxidant capacity [32]. Punicalagin is highly soluble in water, which makes it easier to add to the diluent [33]. However, it has not been thoroughly researched as an antioxidant in ram semen. Therefore, the purpose of this study was to explore the antioxidant effect of punicalagin in protecting sperm from damage caused by ROS during liquid storage at 4 °C.

As shown in Table 1, supplementation with different concentrations of punicalagin improved the motility parameters of ram sperm during liquid storage at 4 °C, particularly in the 30 μM group. After 5 days of storage, the sperm TM and PM of the 30 μM group were significantly higher than those of the other groups. Furthermore, the data indicated that punicalagin treatment was beneficial in maintaining functional integrity, including the integrity of the plasma membrane and acrosome on the 5th day of storage (*p* < 0.05; Table 3). Furthermore, punicalagin also exhibits a concentration-dependent effect. With increasing concentration, sperm TM, PM, plasma membrane, acrosome integrity and kinematic parameters gradually improved by the 5th day of storage (Table 1, Table 2 and Table 3). However, the highest concentration of the additive (45 μM group) did not show a greater protective effect on sperm motility parameters and functional integrity compared to the 30 μM treatment. This suggests that higher concentrations of punicalagin may have a negative effect, reducing its protective effect and potentially causing toxicity to sperm cells [34]. The higher concentrations of antioxidants destroyed the functional integrity of the sperm acrosome and membrane [35,36]. The results showed that the 45 μM group of punicalagin decreased the plasma membrane integrity, acrosome integrity and MMP of sperm compared to the 30 μM group. The 45 μM group may have altered the permeability of the mitochondrial membrane, leading to the release of Cytochrome C from sperm, which affected mitochondrial function and sperm motility [37]. The results are consistent with previous studies. Fedder [38] reported that punicalagin could improve the sperm motility parameters of patients. It was reported that gavage for seven weeks can increase sperm motility, decrease the percentage of abnormal sperm and stimulate spermatogenesis in rats [39]. Mansour [40] reported that feeding punicalagin for six weeks increased sperm motility and concentration while decreasing the rate of abnormal sperm in rats.

Physiological concentrations of ROS contribute to sperm capacitation and the acrosome reaction. However, excessive ROS will have a detrimental effect on the spermatozoa. The sperm’s inherent antioxidant system is insufficient to counteract the excess ROS produced during semen preservation. Excessive ROS can damage the sperm plasma membrane by interacting with polyunsaturated fatty acids (PUFAs), leading to LPO and the production of MDA [41,42]. As shown in Figure 1b,c, the control group exhibited significantly higher levels of ROS and MDA compared to the 15, 30 and 45 μM treatment groups. Punicalagin treatment significantly reduced ROS and MDA content, especially in the 30 μM group. The study showed that punicalagin could alleviate oxidative stress during the preservation of sheep semen at 4 °C. As shown in Figure 1a and Figure 2b,c, the 30 μM treatment group significantly improved sperm SOD activity, CAT activity and TAC activity during liquid storage at 4 °C. The results of the present study showed that adding punicalagin to the diluent increased the activity of antioxidant enzymes and enhanced the TAC of spermatozoa. As a result, the oxidative stress caused by ROS was alleviated, LPO was reduced, MDA production was decreased and sperm quality was ultimately improved. Fouad [43] reported that feeding mice with 15, 30 mg/kg punicalagin decreased the MDA content in the liver. It was reported that punicalagin may alleviate testicular damage caused by oxidative stress by improving the activity of GSH, SOD and CAT [32]. The results are consistent with previous studies. Nrf2 plays an important role in defending against oxidative stress by activating the cellular antioxidant system [44]. Punicalagin may enhance the activity of antioxidant enzymes by activating Nrf2 and regulating the expression of downstream antioxidant genes [45].

Mitochondria play an important role in maintaining sperm fertility, function and signal transduction [15,46]. The mitochondrial OXPHOS is also a significant contributor to ROS production. As shown in Figure 2a, the 30 μM treatment group significantly improved the sperm MMP during liquid storage at 4 °C. These data indicated that punicalagin could protect the normal mitochondrial function of sperm from oxidative stress damage. Hao [47] reported that adding punicalagin to the diluent could enhance the expression of genes associated with the mitochondrial respiratory chain in sperm and maintain normal mitochondrial function in boar semen. This is consistent with the findings of Hao regarding the storage of boar semen at room temperature.

## 5. Conclusions

In conclusion, the *Hu* ram semen diluent containing appropriate concentrations of punicalagin improved the total motility and progressive motility of *Hu* ram sperm, as well as the integrity of the plasma membrane and acrosome, while decreasing the content of ROS and MDA. Furthermore, punicalagin effectively improved TAC activity, MMP, SOD activity and CAT activity during liquid storage at 4 °C. The optimal concentration of punicalagin in the *Hu* ram semen diluent was determined to be 30 μM. The results indicated that a diluent supplemented with punicalagin could improve the quality of ram sperm preserved at 4 °C by increasing antioxidant capacity, mitochondrial potential and reducing oxidative stress.

## Figures and Tables

**Table 1:** Sperm motility parameters affected by five doses of punicalagin.

Motility Parameters	Preserved Period	Different Concentrations (μM)				
		0 (Control)	5	15	30	45
TM (%)	0 d	84.02 ± 1.19 ^A^	83.75 ± 0.44 ^A^	83.68 ± 0.20 ^A^	84.89 ± 1.08 ^A^	84.07 ± 1.00 ^A^
	1 d	81.21 ± 0.04 ^A^	82.35 ± 0.34 ^A^	81.76 ± 0.34 ^AB^	83.12 ± 1.36 ^AB^	81.90 ± 0.17 ^AB^
	2 d	74.79 ± 1.37 ^Bc^	77.04 ± 1.15 ^Bbc^	80.48 ± 0.51 ^Ba^	81.24 ± 0.07 ^Ba^	79.97 ± 1.16 ^Bab^
	3 d	69.25 ± 1.72 ^Cd^	73.42 ± 0.85 ^Cc^	76.82 ± 0.12 ^Cab^	78.46 ± 0.90 ^Ca^	75.19 ± 0.16 ^Cbc^
	4 d	62.57 ± 1.08 ^Dc^	65.88 ± 1.60 ^Dbc^	68.27 ± 0.59 ^Db^	75.16 ± 0.45 ^Da^	68.67 ± 1.15 ^Db^
	5 d	51.09 ± 0.57 ^Ec^	53.33 ± 0.50 ^Ebc^	56.93 ± 2.28 ^Eb^	62.52 ± 1.01 ^Ea^	54.56 ± 0.89 ^Ebc^
PM (%)	0 d	81.44 ± 1.64 ^A^	81.06 ± 0.73 ^A^	80.89 ± 0.56 ^A^	82.58 ± 0.87 ^A^	81.45 ± 0.91 ^A^
	1 d	76.66 ± 0.32 ^Bc^	78.77 ± 0.36 ^Bb^	79.20 ± 0.31 ^Ab^	80.44 ± 0.03 ^ABa^	78.88 ± 0.56 ^Ab^
	2 d	69.76 ± 0.29 ^Cc^	71.06 ± 0.17 ^Cc^	73.86 ± 0.72 ^Bb^	78.18 ± 0.36 ^BCa^	75.26 ± 0.69 ^Bb^
	3 d	61.05 ± 1.35 ^Dd^	67.43 ± 0.77 ^Dc^	69.34 ± 0.50 ^Cbc^	76.01 ± 0.43 ^Ca^	70.16 ± 0.54 ^Cb^
	4 d	56.02 ± 1.07 ^Ed^	59.61 ± 0.64 ^Ec^	64.77 ± 0.91 ^Db^	68.50 ± 0.83 ^Da^	61.04 ± 0.47 ^Dc^
	5 d	39.22 ± 0.22 ^Fc^	40.84 ± 0.25 ^Fc^	43.96 ± 1.14 ^Eb^	50.24 ± 1.58 ^Ea^	44.01 ± 0.83 ^Eb^

Note: Data are expressed as mean ± SEM. Different superscripts (lowercase letter) in the same row (preserved on the same day) show significant differences (*p* < 0.05). Different superscripts (uppercase letter) in the same column (preserved at the same concentration) show significant differences (*p* < 0.05).

**Table 2:** Sperm kinematic parameters affected by five doses of punicalagin.

Kinematic Parameters	Preserved Period	Different Concentrations (μM)				
		0 (Control)	5	15	30	45
VSL (µm/s)	0 d	61.03 ± 2.03 ^A^	61.49 ± 1.08 ^A^	59.71 ± 1.80 ^A^	60.25 ± 2.77 ^A^	61.54 ± 3.39 ^A^
	1 d	35.42 ± 1.27 ^B^	36.06 ± 0.76 ^B^	34.71 ± 1.44 ^C^	38.04 ± 1.53 ^C^	35.41 ± 1.44 ^BC^
	2 d	32.65 ± 1.30 ^BCd^	34.32 ± 1.48 ^BCcd^	38.95 ± 1.60 ^Bb^	44.22 ± 1.23 ^Ba^	37.95 ± 1.18 ^Bbc^
	3 d	32.38 ± 0.53 ^BC^	31.77 ± 0.48 ^CD^	32.52 ± 0.23 ^CD^	33.25 ± 0.63 ^D^	31.54 ± 0.69 ^CD^
	4 d	28.98 ± 0.30 ^CDb^	29.73 ± 0.49 ^Db^	28.90 ± 0.37 ^Db^	31.03 ± 0.28 ^Da^	29.69 ± 0.29 ^Db^
	5 d	27.78 ± 0.99 ^D^	28.68 ± 1.16 ^D^	28.82 ± 0.75 ^D^	30.25 ± 0.61 ^D^	27.71 ± 0.71 ^D^
VCL (µm/s)	0 d	79.31 ± 1.82 ^A^	81.31 ± 1.33 ^A^	78.22 ± 1.61 ^A^	80.12 ± 1.92 ^A^	80.05 ± 2.47 ^A^
	1 d	73.98 ± 1.45 ^B^	71.51 ± 2.37 ^B^	71.39 ± 2.53 ^B^	73.75 ± 0.61 ^B^	72.63 ± 1.68 ^B^
	2 d	71.64 ± 1.55 ^B^	71.61 ± 1.01 ^B^	72.58 ± 1.00 ^B^	73.95 ± 1.32 ^B^	70.32 ± 0.38 ^BC^
	3 d	64.46 ± 0.89 ^Cb^	65.47 ± 1.29 ^Cb^	67.33 ± 2.54 ^BCb^	72.72 ± 1.12 ^Ba^	65.70 ± 0.28 ^CDb^
	4 d	57.90 ± 0.85 ^Dc^	61.14 ± 1.35 ^Cbc^	65.71 ± 1.10 ^Ca^	66.57 ± 0.83 ^Ca^	65.47 ± 2.28 ^CDab^
	5 d	57.77 ± 0.07 ^Db^	61.29 ± 1.55 ^Cab^	61.95 ± 0.87 ^Ca^	64.38 ± 1.62 ^Ca^	61.03 ± 0.31 ^Dab^
VAP (µm/s)	0 d	56.08 ± 1.28 ^A^	57.50 ± 0.94 ^A^	55.31 ± 1.14 ^A^	56.65 ± 1.36 ^A^	56.60 ± 1.74 ^A^
	1 d	50.56 ± 1.68 ^B^	50.48 ± 1.79 ^B^	52.31 ± 1.03 ^AB^	52.15 ± 0.43 ^B^	51.36 ± 1.19 ^B^
	2 d	49.72 ± 0.27 ^B^	50.64 ± 0.71 ^B^	51.32 ± 0.71 ^B^	52.29 ± 0.93 ^B^	50.65 ± 1.10 ^B^
	3 d	45.58 ± 0.63 ^Cb^	46.30 ± 0.91 ^Cb^	47.61 ± 1.80 ^Cab^	51.42 ± 0.79 ^Ba^	46.29 ± 1.61 ^Cb^
	4 d	40.85 ± 0.05 ^Dc^	43.23 ± 0.95 ^Cb^	46.46 ± 0.78 ^CDa^	47.07 ± 0.59 ^Ca^	46.46 ± 0.20 ^Ca^
	5 d	40.94 ± 0.6 ^Db^	43.33 ± 1.10 ^Cab^	43.80 ± 0.62 ^Da^	45.52 ± 1.15 ^Ca^	43.15 ± 0.22 ^Cab^
MAD (°/s)	0 d	251.35 ± 10.33 ^A^	255.93 ± 11.96 ^A^	264.13 ± 3.08 ^A^	261.05 ± 10.49 ^A^	258.43 ± 6.62 ^A^
	1 d	136.86 ± 1.62 ^Bc^	144.63 ± 1.88 ^Bc^	165.85 ± 8.75 ^Bb^	195.04 ± 3.77 ^Ba^	169.35 ± 3.76 ^Bb^
	2 d	130.35 ± 1.53 ^Bb^	138.77 ± 8.80 ^Bb^	136.80 ± 5.65 ^Cb^	157.98 ± 0.75 ^Ca^	130.60 ± 0.21 ^Cb^
	3 d	85.52 ± 3.88 ^Cc^	88.46 ± 5.24 ^Cc^	104.10 ± 1.85 ^Db^	125.97 ± 2.82 ^Da^	105.05 ± 5.22 ^Db^
	4 d	82.39 ± 2.37 ^Cb^	84.83 ± 0.87 ^Cb^	93.85 ± 3.52 ^Dab^	104.65 ± 5.19 ^Ea^	87.67 ± 3.85 ^Eb^
	5 d	45.74 ± 0.61 ^Dc^	54.69 ± 1.55 ^Db^	59.44 ± 2.46 ^Eb^	75.24 ± 4.80 ^Fa^	58.36 ± 2.78 ^Fb^

Note: Data are expressed as mean ± SEM. Different superscripts (lowercase letter) in the same row (preserved on the same day) show significant differences (*p* < 0.05). Different superscripts (uppercase letter) in the same column (preserved at the same concentration) show significant differences (*p* < 0.05).

**Table 3:** Sperm plasma membrane and acrosome integrity affected by five doses of punicalagin.

Parameters	Preserved Period	Different Concentrations (μM)				
		0 (Control)	5	15	30	45
Plasma membrane (%)	0 d	86.24 ± 0.45 ^A^	86.00 ± 0.66 ^A^	86.62 ± 1.58 ^A^	86.29 ± 0.61 ^A^	86.07 ± 0.95 ^A^
	1 d	80.44 ± 0.52 ^Bc^	81.65 ± 0.44 ^Bbc^	83.55 ± 0.97 ^Bab^	85.06 ± 0.61 ^ABa^	82.64 ± 0.35 ^Bb^
	2 d	75.10 ± 0.91 ^Cd^	78.49 ± 0.25 ^Cc^	80.48 ± 0.43 ^Cb^	83.56 ± 0.63 ^Ba^	79.22 ± 0.35 ^Cbc^
	3 d	70.35 ± 0.63 ^Dd^	74.46 ± 0.38 ^Dc^	77.32 ± 0.34 ^Db^	80.21 ± 0.33 ^Ca^	76.40 ± 0.67 ^Db^
	4 d	66.10 ± 0.89 ^Ed^	69.52 ± 0.52 ^Ec^	72.42 ± 0.75 ^Eb^	77.02 ± 0.51 ^Da^	72.44 ± 1.07 ^Eb^
	5 d	60.50 ± 0.75 ^Fd^	65.12 ± 1.05 ^Fc^	69.16 ± 0.42 ^Fb^	73.98 ± 0.63 ^Ea^	65.68 ± 0.94 ^Fc^
Acrosome integrity (%)	0 d	90.17 ± 0.43 ^A^	89.75 ± 0.36 ^A^	90.15 ± 0.62 ^A^	91.12 ± 0.82 ^A^	90.63 ± 0.27 ^A^
	1 d	85.93 ± 0.44 ^Bc^	86.32 ± 0.38 ^Bbc^	87.99 ± 0.66 ^Bab^	89.70 ± 0.71 ^Aa^	88.29 ± 0.74 ^Bab^
	2 d	83.37 ± 0.26 ^Cc^	83.71 ± 0.34 ^Cc^	86.11 ± 0.74 ^Cb^	87.82 ± 0.70 ^Ba^	85.10 ± 0.48 ^Cbc^
	3 d	81.15 ± 0.70 ^Dc^	82.34 ± 0.71 ^Cbc^	83.56 ± 0.39 ^Db^	86.67 ± 0.55 ^BCa^	82.37 ± 0.69 ^Dbc^
	4 d	78.69 ± 0.53 ^Ec^	78.65 ± 0.62 ^Dc^	81.00 ± 0.26 ^Eb^	85.35 ± 0.34 ^Ca^	81.80 ± 0.45 ^Db^
	5 d	75.77 ± 0.41 ^Fd^	77.64 ± 0.78 ^Dc^	79.84 ± 0.27 ^Eb^	82.75 ± 0.22 ^Da^	79.09 ± 0.51 ^Ebc^

Note: Data are expressed as mean ± SEM. Different superscripts (lowercase letter) in the same row (preserved on the same day) show significant differences (*p* < 0.05). Different superscripts (uppercase letter) in the same column (preserved at the same concentration) show significant differences (*p* < 0.05).

## Data Availability

All data sets collected and analyzed during the current study are available from the corresponding author on reasonable request.
